# Highly sensitive and multiplex detection of nine potential bioterrorism viral agents in a single reaction by multiplex probe amplification (MPA) with melting curve analysis

**DOI:** 10.1128/spectrum.01078-25

**Published:** 2025-07-31

**Authors:** Yuchang Li, Jianping Wang, Yuan Huang, Xueping Ma, Haiping Wu, Sen Zhang, Fuli Tan, Yuehong Chen, Jing Li, Ye Feng, Xiaokun Li, Guohua Zhou, Tao Jiang, Xiaoping Kang

**Affiliations:** 1 State Key Laboratory of Pathogen and Biosecurity, The Academy of Military Medical Sciences, Beijing, China; 2 R&D Department, Guangzhou Biotron Biotechnology Co., Ltd., Guangzhou, Guangdong, China; 3 Department of Clinical Pharmacy, Jinling Hospital, Affiliated Hospital of Medical School, State Key Laboratory of Analytical Chemistry for Life Science, Nanjing University 630794, Nanjing, Jiangsu, China; Children's National Hospital, Washington, DC, USA

**Keywords:** virus, biothreat virus, multiplex detection, single reaction, multiplex probe amplification with melting curve analysis

## Abstract

The rapid and accurate detection of highly pathogenic viruses is critical for public health, especially in the context of potential bioterrorism threats. This study presents a novel multiplex probe amplification (MPA) assay combined with melting curve analysis for the simultaneous detection of nine high-threat viral agents: Ebola virus, Lassa virus, Crimean-Congo hemorrhagic fever virus, Rift Valley fever virus, Chikungunya virus, Monkeypox virus, Eastern equine encephalitis virus, Tick-borne encephalitis virus, and Venezuelan equine encephalitis virus. The MPA-nine-viruses assay utilizes three fluorescence channels and unique melting temperatures to differentiate between targets, achieving high sensitivity with a limit of detection as low as 1.5–15 copies/μL. The MPA-nine-viruses was highly specific and distinguished mixed targets accurately, even for the nine viruses’ mixture. The assay was validated using 392 simulated samples (including serum, swabs, mosquito vectors, and soil samples) and 22 clinical samples, demonstrating 100% accuracy. The MPA-nine-viruses assay offers a cost-effective, high-throughput solution for large-scale screening and is poised to play a crucial role in the prevention and control of outbreaks and bioterrorism events involving these pathogens.

**IMPORTANCE:**

Ebola virus, Lassa virus, Crimean-Congo hemorrhagic fever virus, Rift Valley fever virus, Chikungunya virus, Monkeypox virus, Eastern equine encephalitis virus, Tick-borne encephalitis virus, and Venezuelan equine encephalitis virus can cause severe infections and diseases, such as hemorrhagic fever, encephalitis, meningitis, and chikungunya fever. These viruses pose significant threats to public health due to their high infectivity and mortality rates, and they could potentially be used as bioterrorism agents. Early detection is the most critical step for effective prevention and control of infection. However, there is no sensitive nucleic acid detection method for the nine high-threat viral agents tested simultaneously. In this study, we developed multiplex probe amplification (MPA) with Melting Curve PCR method, named MPA-nine-viruses, which overcame the limitation of the available fluorescence channel number and realized simultaneous detection of nine high-threat viral agents in a single reaction, with high sensitivity and specificity for the targets.

## INTRODUCTION

Ebola virus (EBOV), Lassa virus (LASV), Crimean-Congo hemorrhagic fever virus (CCHFV), Rift Valley fever virus (RVFV), Chikungunya virus (CHIKV), Monkeypox virus (MPXV), Eastern equine encephalitis virus (EEEV), Tick-borne encephalitis virus (TBEV), and Venezuelan equine encephalitis virus (VEEV) can cause severe infections and diseases, such as hemorrhagic fever, encephalitis, meningitis, and chikungunya fever. These viruses are highly infectious and can induce high mortality rates (1–6). In the Biological Agent Biosafety Category Lists, most of them are classified as the highest or second-highest threat levels by the European Union and the U.S. Centers for Disease Control and Prevention (CDC) (7, 8).

Over the past two decades, there have been multiple outbreaks of these viral infectious diseases. For example, since 2000, chikungunya fever has frequently erupted in affecting regions including the Democratic Republic of Congo, Indonesia, Singapore, the Philippines, and Cambodia. Between 2014 and 2016, chikungunya fever caused 1.9 million suspected cases in the Americas, spanning over 50 countries and regions, with 267 reported deaths (9–11). From 2014 to 2016, a severe Ebola outbreak occurred in Guinea, Liberia, and Sierra Leone, resulting in over 28,000 infections and more than 11,300 deaths (12, 13). In 2022, an monkeypox outbreak spread across Europe and multiple countries worldwide (13–15).

These viruses pose significant threats to public health due to their high infectivity and mortality rates, and they could potentially be used as bioterrorism agents. In some cases, these highly pathogenic viruses can cause similar or identical clinical symptoms. Since there are no available treatments for these viruses to date, early detection is the most critical step for effective prevention and control of infection (16–18). To address future outbreaks and potential biothreat attacks by these highly pathogenic viruses, the public health system requires an effective detection method capable of accurately identifying these pathogens in complex samples. A multiplex, efficient, and cost-saving detection method is in great demand.

Currently, the main types of nucleic acid testing (NAT) can be classified into three groups: hybridization, polymerase chain reaction (PCR), and sequencing (19). The conventional multi-fluorescence channel qPCR, which relies on various fluorescent probes for multiple target detection, is the most commonly used method in clinical settings (20). However, it is limited by the number of available fluorescence channels. Typically, a single channel can only detect one target, restricting the number of pathogen targets to a maximum of four to six per reaction. Clinical samples often require the detection of numerous pathogen targets, necessitating multiple reactions to distinguish each potential pathogen, which increases the complexity and difficulty of clinical testing (21).

Microfluidic qPCR uses fluidic circuits to channel nanoliters of samples and assay components into multiple microscopic reaction chambers for amplification and detection. This method can detect up to 23 targets in a single reaction, offering high throughput and efficiency. However, it is limited to one sample per reaction, making it unsuitable for large-scale screening (22).

Multiplex probe amplification (MPA) with melting curve PCR was developed as an alternative biomarker amplification method that overcomes these limitations. The technology employs specially designed fluorescent probe hybrids (THO:PCO), where each probe hybrid has a unique melting temperature (*T*_m_) based on its specific nucleotide composition and length. Different melting temperatures correspond to specific detection targets, and when combined with four fluorescence channels, the temperature and fluorescence create a two-dimensional locking relationship that identifies specific targets. With optimized primer-probe combinations and detection conditions, MPA can theoretically detect dozens of target genes in a single reaction (22, 23).

**Fig 1:**
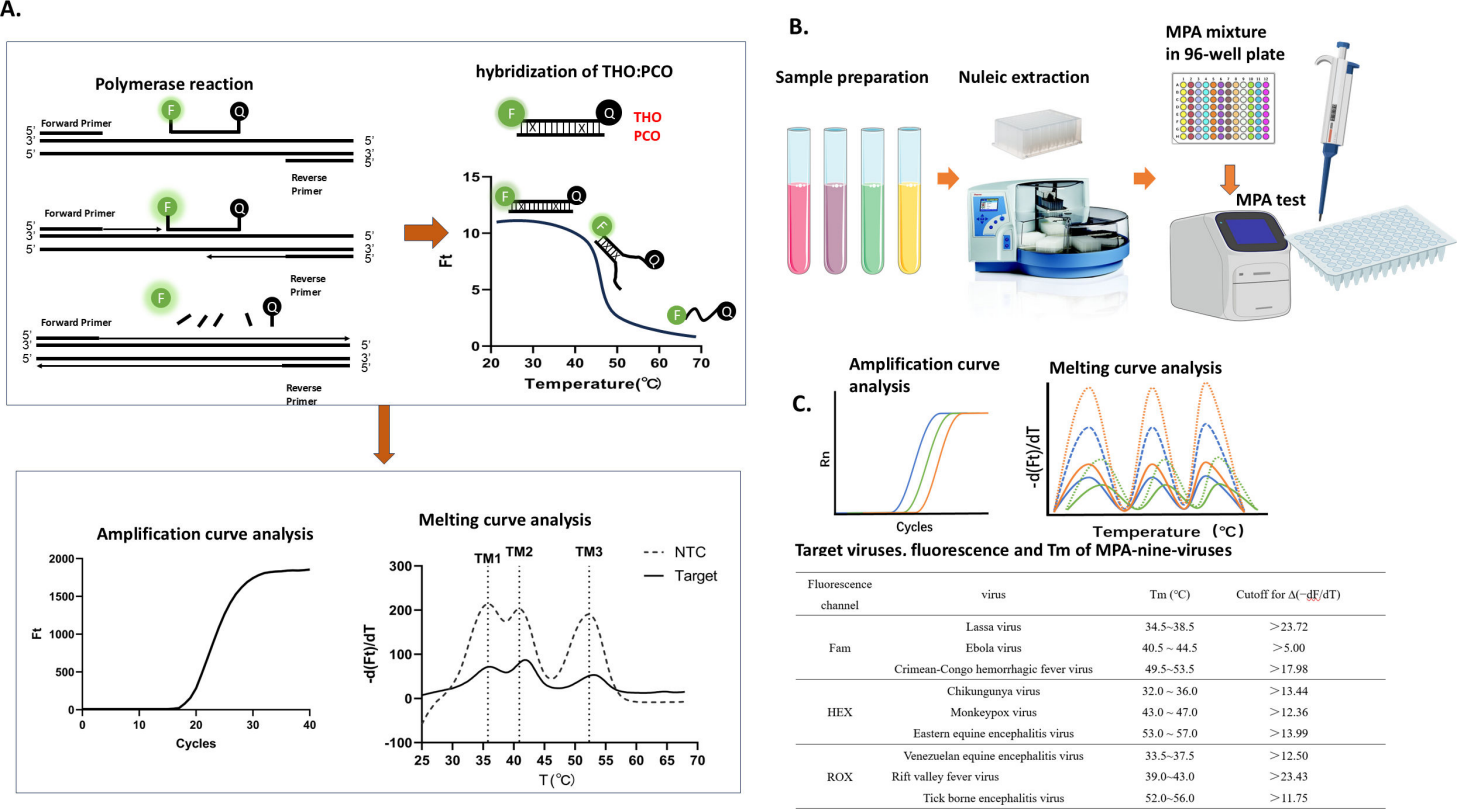
Illustrating the multiplex probe amplification (MPA) with melting curve analysis for nine high pathogenic viruses. (A) Description of the mechanism of MPA. (B) Workflow diagram illustration of MPA. (C) Result analysis of MPA-nine-viruses.

As shown in Fig. 1A, when a target template is present in the sample, the THO probe binds to the target template and is hydrolyzed by DNA polymerase, generating an amplification curve. Since the THO is consumed, it cannot form a THO/PCO hybrid double-stranded structure, resulting in either no melting peak or a weak melting peak. In this case, the melting peak of the sample containing the target template shows a significant melting peak difference compared to the negative control, allowing for the identification of the specific pathogen.

This technology has been applied for the detection of six fungal pathogens and eight enteroviruses (24, 25). However, there have been no reports of its use for detecting the high pathogenic viruses.

In this study, we successfully developed a multiplex method using MPA with melting curve fluorescent array technology for the detection of nine bioterrorism-related viral targets, named MPA-nine-viruses. The schematic diagram is shown in Fig. 1B and C, which enables highly specific and sensitive detection of target pathogens, including Ebola virus, Lassa virus, Crimean-Congo hemorrhagic fever virus, Rift Valley fever virus, Chikungunya virus, MPXV, Eastern equine encephalitis virus, Tick-borne encephalitis virus, and Venezuelan equine encephalitis virus. The MPA-nine-viruses demonstrated outstanding performance on various complex simulated samples, including human serum, swabs, mosquito vectors, and soil. Additionally, clinical samples were validated.

## MATERIALS AND METHODS

### Target sequences selection

The genome sequences of the nine target viruses were obtained from the GenBank database. For each virus species, 10 different strain genome sequences were selected. These sequences were aligned using MegAlign, and conserved fragments were identified as target genes. The selected target sequences for each virus are listed in Table S1.

### Primer and probe design

For the MPA assay, the primers were similar to those used for traditional qPCR. The MPA probes consist of two partially complementary oligonucleotides: the dual-labeled target-hybridizing oligonucleotide (THO) and a corresponding partially complementary oligonucleotide (PCO). Each THO is fully complementary to the target sequences, identical to those used in traditional qPCR, while the PCO sequences were engineered to have several nucleotide mismatches against their corresponding THO sequences.

Using primer and probe design software (Oligo 7.0 and Primer Premier 6.0), multiplex qPCR amplification primers and THO probes were designed for each target viral sequence. The design principles were as follows: (i) primer/probe length: 18–25 bp; (ii) melting temperature (*T*_m_) of primer pairs: 54–64°C; (iii) *T*_m_ of THO probes: 59–79°C; (iv) amplicon length: 100–150 bp; (v) maximum Δ*G* of hairpin structures for primers: ≤6.0 kcal/mol; and (vi) maximum Δ*G* of primer dimers in each group: ≤10.0 kcal/mol.

Primer and probe specificity was verified using the BLAST search tool to ensure they matched the target sequences without cross-reactivity to non-target sequences. The hairpin structures, dimer formations, GC content, and theoretical Tm values of the primers and probes were analyzed to ensure compliance with the design criteria.

Two to five primer-probe (THO) combination sets for each target virus were obtained as candidate sets and synthesized by Sangon Biotech (Shanghai, China). Additionally, internal control primers and THO probe targeting conserved sequences in the human genome were designed, with the THO probe labeled with a Cy5 reporter group.

Using a certain amount of target plasmids as templates, quantitative PCR was performed for each primer-THO probe combination, and the primer-THO probe combinations with lowest Ct were selected as the most sensitive primer set for MPA-nine-viruses configuration. The THO probes were labeled with different fluorescent reporter groups (FAM/HEX/ROX) at their 5′ ends, and quenching group BHQ at their 3′ ends. The sequences of the selected primer-probe are shown in Table S2. Sequence alignment analysis revealed that each primer-probe set had good sequence consistency with the target sequences from different strains.

Then, the PCO probes were designed to produce specific melting temperatures by introducing several nucleotide mismatches against their corresponding THO sequences. For each selected THO probe, a reverse complementary sequence with partial base modifications was designed to create the PCO probe. The THO probe can hybridize with the PCO probe to form a dimer with a specific *T*_m_, enabling the differentiation of multiple target genes within the same fluorescence channel based on their distinct *T*_m_ values. The PCO probes were synthesized with phosphate blocks at 3′ ends. The fluorescence channel and specific *T*_m_ value for each pathogen target are shown in Fig. 1C. The sequences of selected PCO probes are listed in Table S2.

### Virus-like particles (VLPs) preparation

The target sequences listed in Table S1 were synthesized by Sangon Biotech (Shanghai, China) and inserted into the plasmid pE380-MS2. The recombinant plasmid was transformed into *Escherichia coli BL21*, and positive clones were cultured in LB medium containing ampicillin and cultured at 37°C, with shaking. At 180 rpm for 4–6 hs until the OD_600_ reached 0.6-0.8, 1 mM of Isopropyl β-d-1-thiogalactopyranoside was added to induce the VLPs production. After shaking at 180 rpm for 16 h at 16°C, the culture was centrifuged, and the bacterial pellet was collected. The pellet was sonicated on ice, and the sonicated product was centrifuged at 9,000 rpm for 30 min. The supernatant was collected into a 15 mL centrifuge tube, containing the VLPs.

### Nucleic acid extraction

Nucleic acids were extracted from 200 µL of each sample or viral culture using the Viral RNA Mini Kit (QIAGEN, Hilden, Germany). A total of 50 µL of nucleic acid was eluted from each extraction column for each sample.

### qPCR assay

The qPCR assay was used to verify clinical samples and VLPs. The qPCR reaction mixture contained 5 µL of RNA, 5 µL of 4× TaqMan Fast Virus 1-Step Mix (Applied Biosystems, Vilnius, Lithuania), 1 µL of forward primer (10 µM), 1 µL of reverse primer (10 µM), 0.5 µL of probe (10 µM), and 7.5 µL of sterile deionized water, with a final volume of 20 µL.

Reactions were performed on a Bio-Rad CFX96 Real-Time PCR instrument (Bio-Rad Laboratories Co., Ltd., California). The amplification conditions were as follows: reverse transcription at 50°C for 5 min; pre-denaturation at 95°C for 10 seconds; and 40 cycles of PCR amplification consisting of denaturation at 95°C for 5 s, annealing at 60°C for 30 s, and fluorescence measurement (25).

### Droplet digital PCR (ddPCR)

The TILLA Naica Crystal ddPCR System was employed to precisely measure the concentration of the nine pathogenic viruses. A series of 10-fold dilutions of nucleic acids extracted from virus cultures or VLPs were prepared. The reaction mixture consisted of 12.5 µL of qScript XLT One-Step RT-qPCR ToughMix (QuantaBio), 2.5 µL of fluorescein sodium salt (1 µM), 2.5 µL of forward primer (10 µM), 2.5 µL of reverse primer (10 µM), 0.625 µL of probe (10 µM), 1.875 µL of sterile deionized water, and 2.5 µL of nucleic acid, with a final volume of 25 µL.

The mixture was loaded into Sapphire chips and placed into the naica Geode, a combined droplet generator and thermocycler, following the manufacturer’s instructions. The amplification program consisted of 3 min at 95°C for denaturation, followed by 60 cycles of 15 s at 95°C and 3 s at 62°C. After amplification, the Sapphire chips were scanned using the naica Prism3 system with Crystal Reader v4.0 (Stilla Technologies) software for fluorophore visualization. Target concentrations and confidence intervals were automatically calculated by Crystal Miner v4.0 based on Poisson distribution (26).

### MPA assay

The MPA reaction mixture contained an enzyme mixture, a working solution, and ddH_2_O. The enzyme mixture consisted of DNA polymerase (2.5 U), Mg^2+^ (1.5 mmol/L), and dNTPs (200 µmol/L), while the working solution contained primers and probes (Table 1). By optimizing the concentration of each primer and probe, as well as the amplification procedure, the optimal reaction mixture was obtained.

**TABLE 1:** Amount of each primer/probe in working solution

Primer/probe	Concentration (µM）	Volume (µL）
LASV-F	10	0.22
LASV-R	10	0.22
LASV-THO	10	0.11
LASV-PCO	10	0.22
XHFV-F	10	0.11
XHFV-R	10	0.11
XHFV-THO	10	0.075
XHFV-PCO	10	0.15
EBO-Zaire-F	10	0.0275
EBO-Zaire-R	10	0.0275
EBO-Zaire-THO	10	0.055
EBO-Zaire-PCO	10	0.11
CHV-F	10	0.11
CHV-R	10	0.11
CHV-THO	10	0.055
CHV-PCO	10	0.11
mpox-F	10	0.055
mpox-R	10	0.055
mpox-THO	10	0.055
mpox-PCO	10	0.11
EEEV-F	10	0.11
EEEV-R	10	0.11
EEEV-THO	10	0.11
EEEV-PCO	10	0.22
TBEV-F	10	0.088
TBEV-R	10	0.088
TBEV-THO	10	0.055
TBEV-PCO	10	0.11
RVFV-F	10	0.088
RVFV-R	10	0.088
RVFV-THO	10	0.055
RVFV-PCO	10	0.11
VEEV-F	10	0.055
VEEV-R	10	0.055
VEEV-THO	10	0.033
VEEV-PCO	10	0.066
gDNA-F	10	0.0075
gDNA-R	10	0.0075
gDNA-THO1	10	0.0195
gDNA-THO2	10	0.0375
gDNA-PCO	10	0.0405
H2O		0.3515
Total		4

Amplification was performed on a SLAN-96S Real-Time PCR instrument (Hongshi Tech, China) under the following conditions: reverse transcription at 55°C for 10 min; pre-denaturation at 95°C for 3 min; and 42 cycles of PCR amplification consisting of denaturation at 95°C for 10 s, annealing at 60°C for 45 s, and fluorescence measurement, followed by extension at 69°C for 20 s. Melting curve analysis was performed as follows: 45°C for 20 s, 35°C for 20 s, 25°C for 1 min, and continuous fluorescence measurement from 25°C to 68°C.

Results were identified by amplification curve analysis and melting curve analysis. One target virus corresponds to a certain temperature at a certain fluorescence channel, as listed in Fig. 1C. Amplification curve with CT <36 was used to determine positivity; the melting curve analysis was used to determine the virus species of the positive samples. The melting curve peak reduction values (∆(−dF/dT)) at each Tm were calculated, and these were considered positive when the ∆(−dF/dT) was higher than the cutoff (Fig. 1C).


$$\Delta \left(-dF/dT\right)=\;\left(-\frac{dF}{dT}\right)\text{negative}-\;\left(-\frac{dF}{dT}\right)\text{sample}$$

### Simulated samples

Inactivated viruses, including Chikungunya virus, Tick-borne encephalitis virus, MPXV, Dengue virus, Japanese encephalitis virus, Yellow fever virus, and Vaccinia virus, as well as VLPs, including VLPs of Ebola virus, Lassa fever virus, Rift Valley fever virus, Eastern equine encephalitis virus, Venezuelan equine encephalitis virus, Crimean-Congo hemorrhagic fever virus, Western equine encephalitis virus, Machupo virus, and Bunya virus, were used to prepare the simulated samples; Dengue virus, Japanese encephalitis virus, Yellow fever virus, Vaccinia virus, VLPs of Western equine encephalitis virus, Machupo virus, and Bunya virus were used as non-targets for specificity validation.

Four types of simulated samples were prepared using media of human serum, swab sampling solution, soil solution (1 mg/mL soil in PBS), and mosquito cells (10^5^ C6/36 cells/mL in PBS). A total of 72 single-pathogen simulated samples were prepared by spiking middle (5 × 10^4^ gene copies/mL) or low (5 × 10^3^ gene copies/mL) concentrations of cultured virus or VLPs into the respective media, eight samples for each target virus. Additionally, 288 dual-virus simulated samples were prepared to verify the accuracy of MPA-nine-viruses for two viruses’ co-infection.

Non-target viruses or VLPs were also spiked into the four types of media at 1 × 10^6^ gene copies/mL to prepare 32 negative samples. In total, 392 samples were prepared. All samples were randomized to code for nucleic acid extraction and MPA test.

### Clinical samples

Nucleic acid extracted from 10 swab samples of mpox patients was obtained from the Jiangsu Provincial Center for Disease Control and Prevention, with qPCR-confirmed positivity. Two tick-borne encephalitis virus (TBEV)-positive serum samples were obtained from Mudanjiang Forestry Central Hospital, confirmed by qPCR and antibody testing. Two healthy human serum samples, two healthy human swab samples, four influenza virus-positive throat swab samples, and two COVID-19-positive throat swab samples were obtained from the General Hospital of the People’s Liberation Army.

## RESULTS

### Preparation and identification of the VLPs

Since the nine viruses targeted in this study are all highly pathogenic and the active strains were difficult to obtain, we prepared VLPs as targets for primer selection, MPA optimization, sensitivity, specificity evaluation, and simulated sample preparation.

**Fig 2:**
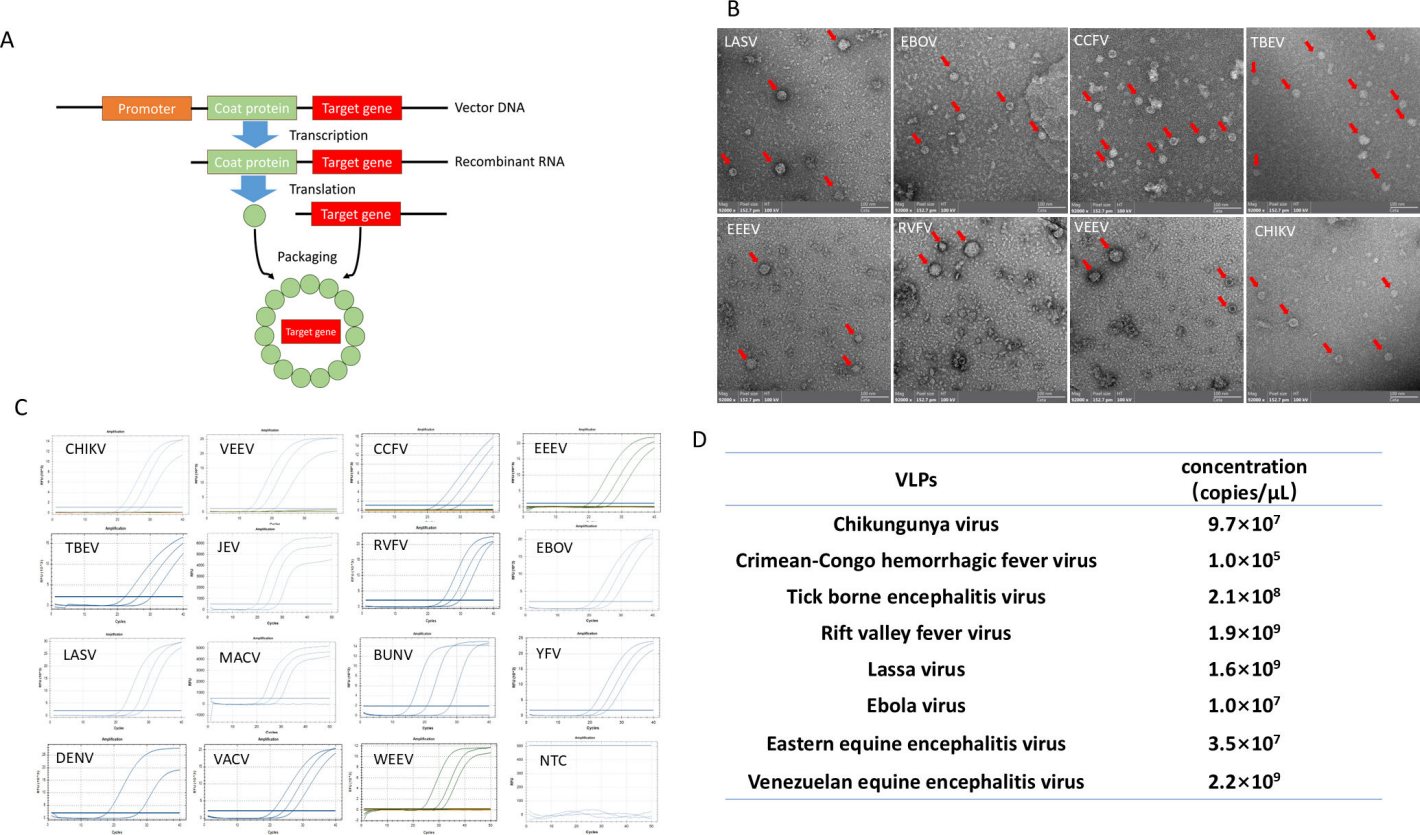
Preparation of virus-like particles. (A) Diagram illustration of VLP preparation. (B) TEM analysis of VLP. (C) qPCR analysis of VLP. (D) Concention identification of VLP by digital droplet qPCR. CCHFV, Crimean-Congo hemorrhagic fever virus; CHIKV, Chikungunya virus; EBOV, Ebola virus; EEEV, Eastern equine encephalitis virus; LASV, Lassa virus; NTC, negative control; RVFV, Rift Valley fever virus; TBEV, Tick-borne encephalitis virus; VEEV, Venezuelan equine encephalitis virus.

The target genes were inserted into the plasmid pE380-MS2 to produce the VLPs (Fig. 2A). By negative staining and observing under an electron microscope, the harvested VLPs were visualized with regular morphology and distribution, and approximately 30 nm in diameter (Fig. 2B), which was consistent with the expected size. To verify whether the target genes were encapsulated in the VLPs, nucleic acids were extracted from the VLPs and subjected to qPCR amplification, which confirmed positive signals for the target gene amplification, indicating that the target viral genes were correctly encapsulated in the VLPs (Fig. 2C).

Furthermore, digital PCR was used to quantify the concentration of VLP genes, the concentration of the VLPs was 10^7^–10^8^ gene copies/µL, confirming the high efficiency of the VLPs production and correct encapsulation of the targets in VLPs (Fig. 2D).

### The MPA-nine-viruses assay demonstrated high sensitivity and specificity for the targets

The selected primers, THO, and PCO probes for the nine target viruses were combined for MPA-nine-viruses. To improve the detection sensitivity, the concentration of each primer and probe in the reaction mixture was optimized by adjusting in the range of 10–100 nmol/L, and the final concentrations of each primer or probe used in MPA-nine-viruses were listed in Table 2.

**TABLE 2:** Information of multi-virus samples and MPA results^*a*^

Sample	MPA results							Viruses contained
	FAM		HEX		ROX		Viruses identified	
	Ct	*T*_m_	Ct	*T*_m_	Ct	*T*_m_		
1	NoCt	/	29.60	53.50	29.89	35.00	EEEV, TBEV	EEEV, TBEV
2	27.48	51.00	32.02	53.50	NoCt	/	CCFV, EEEV	CCFV, EEEV
3	27.27	51.00	NoCt	/	34.03	35.00	CCFV, TBEV	CCFV, TBEV
4	26.64	42.00	32.55	34.00	NoCt	/	EBOV, CHIKV	EBOV, CHIKV
5	NoCt	/	31.55	44.00	33.41	35.00	MPXV, TBEV	MPXV, TBEV
6	NoCt	/	30.79	34.00	31.88	51.50	CHIKV, RVFV	CHIKV, RVFV
7	23.22	35.83	25.09	44.78	23.68	35.56	LASV, EEEV, TBEV	LASV, EEEV, TBEV
8	19.38	35.83; 52.38	22.30	44.78	18.83	35.56; 52.99	LASV, CCFV, EEEV, TBEV, RVFV	LASV, CCFV, EEEV, TBEV, RVFV
9	22.10	35.83; 52.38	23.70	34.03; 54.32	21.17	35.56; 41.36; 52.99	LASV, CCFV, CHIKV, EEEV, TBEV, VEEV, RVFV	LASV, CCFV, CHIKV, EEEV, TBEV, VEEV, RVFV
10	19.99	36.32; 42.88; 51.85	21.19	34.03; 45.14; 54.32	19.17	35.56; 41.36; 52.99	LASV,EBOV,CCFV, CHIKV,MPXV,EEEV, TBEV,VEEV,RVFV	LASV,EBOV, CCFV, CHIKV,MPXV,EEEV, TBEV,VEEV,RVFV

^*a*^ "/" indicates that no *T*_m_ with significantly reduced melting peak.

**Fig 3:**
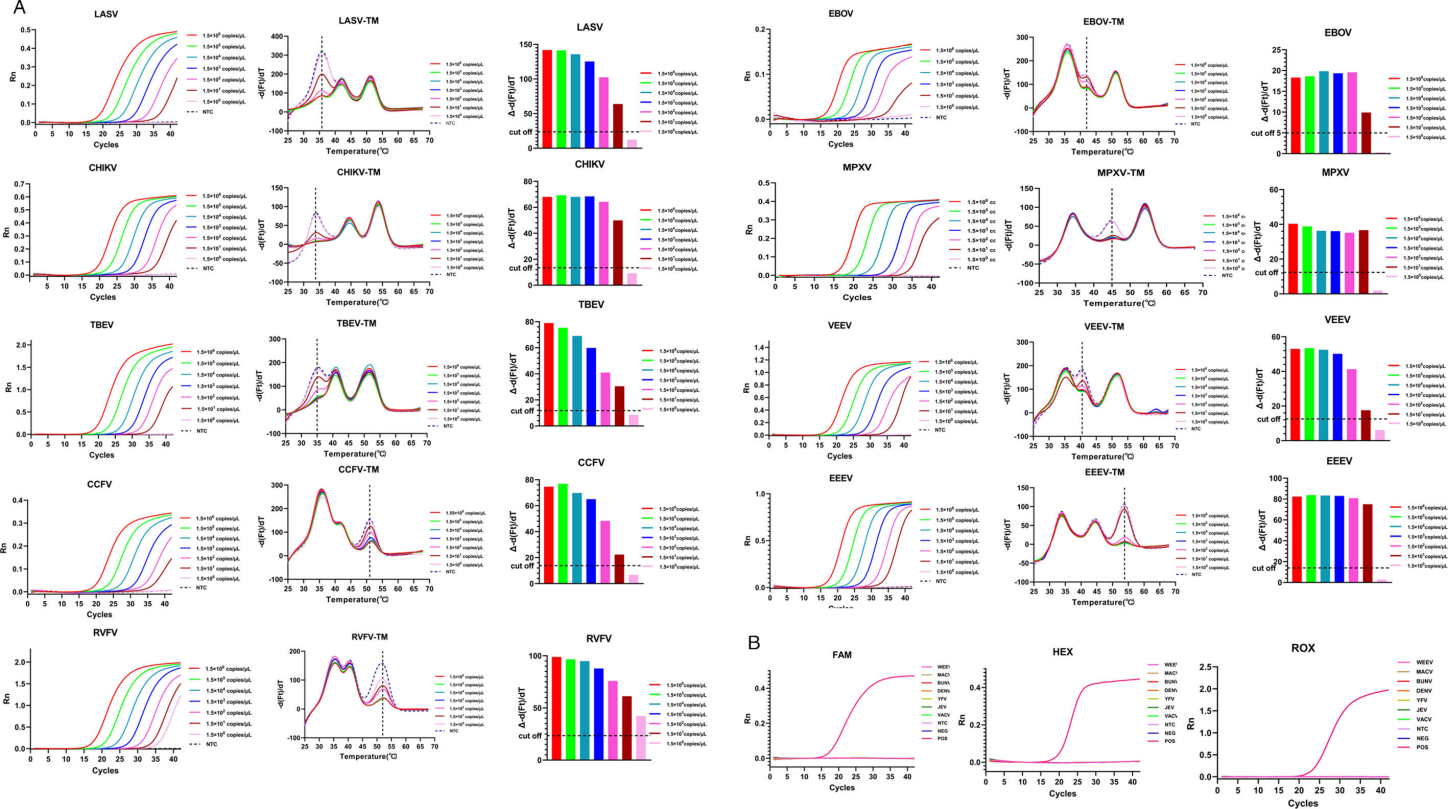
Sensitivity and specificity of MPA-nine-viruses. A limit of detection of MPA-nine-viruses for each target. Specificity of MPA-nine-viruses for non-target viruses. Bunv, Bunyavirus; CCHFV, Crimean-Congo hemorrhagic fever virus; CHIKV, Chikungunya virus; DENV, Dengue virus; EBOV, Ebola virus; EEEV, Eastern equine encephalitis virus; JEV, Japanese encephalitis virus; LASV, Lassa virus; MACV, Machupo virus; MPXV, Monkeypox virus; NTC, negative control; POS, positive control; RVFV, Rift Valley fever virus; TBEV, Tick-borne encephalitis virus; VACV, Vaccinia virus; VEEV, Venezuelan equine encephalitis virus; WEEV, Western equine encephalitis virus; YFV, Yellow fever virus.

The limit of detection (LOD) for the MPA-nine-viruses was determined using serially diluted nucleic acids of VLPs. The nucleic acid was diluted 10-fold (0.15–1.5 × 10^6^ copies/μL) as templates, with three replicates per sample for MPA amplification. The amplification curves and melting curve amplification were shown in Fig. 3A. The results showed that the LOD of RVFV reached 1.5 copies/μL, LODs for other eight viruses reached 15 copies/μL, indicating that the MPA-nine-viruses is highly sensitive for detecting the nine target viral nucleic acids. It is noteworthy that the positive signal was only observed in the corresponding detection channels, and the significantly decreased melting curve peak was at the specific temperature, which was specific to the target virus. The other melting curve peaks were similar to that of the negative control, demonstrating no cross-amplification among the nine target viral genes.

The specificity of the MPA-nine-viruses was further verified using seven unrelated viral nucleic acids (Dengue virus, Japanese encephalitis virus, Yellow fever virus, vaccinia virus, VLPs of Western equine encephalitis virus, Machupo virus, and Bunya virus), all of which yielded no positive signals (Fig. 3B), confirming the high specificity of the MPA-nine-viruses.

### MPA-nine-viruses accurately detected mixed virus samples

**Fig 4:**
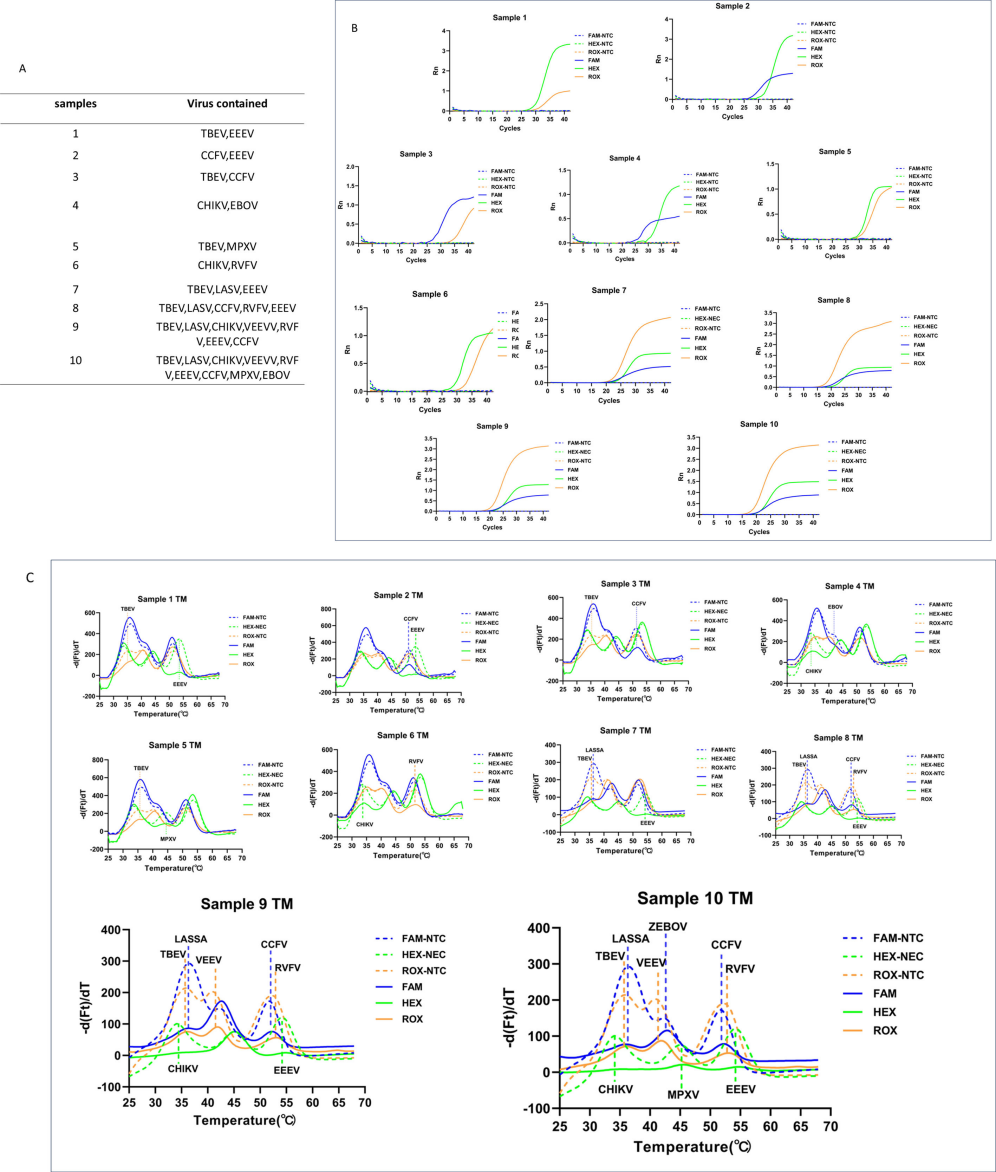
Evaluation of MPA-nine viruses by mixed virus samples. (A) Information of the 10 mixed samples. (B) Amplification results of the mixed virus samples. (C) Melting curve analysis of the mixed viral samples. CCHFV, Crimean-Congo hemorrhagic fever virus; CHIKV, Chikungunya virus; EBOV, Ebola virus; EEEV, Eastern equine encephalitis virus; LASV, Lassa virus; MPXV, Monkeypox virus; RVFV, Rift Valley fever virus; TBEV, Tick-borne encephalitis virus; VEEV, Venezuelan equine encephalitis virus. The negative control was in dotted line, and the samples were in solid.

To address potential mixed infections or multi-virus attacks in the future, we evaluated the performance of the MPA-nine-viruses method in detecting mixed virus samples. The genes of the nine target viruses were randomly mixed in combinations of 2, 3, 5, 7, and 9 viruses, resulting in a total of 10 mixed virus samples. Nucleic acids were extracted and subjected to MPA detection. As shown in Fig. 4 and Table 2, the targets were detected out in all 10 mixed virus samples.

Notably, sample 10, which contained nucleic acids from all nine target viruses, showed positive signals for all nine targets. Positive amplification curves appeared at three fluorescence channels of FAM, HEX, and ROX. In each channel, the melting curve peak significantly reduced at three distinct Tm values, and all of the peak reduction values (∆(−dF/dT)) were higher than the cutoff (Fig. 4C). This result demonstrated the accurate amplification of this MPA-nine-viruses for multiple virus mixtures in a single reaction, without competitive inhibition, even in the presence of as many as nine targets.

### MPA-nine-viruses accurately detects complex simulated samples

**Fig 5:**
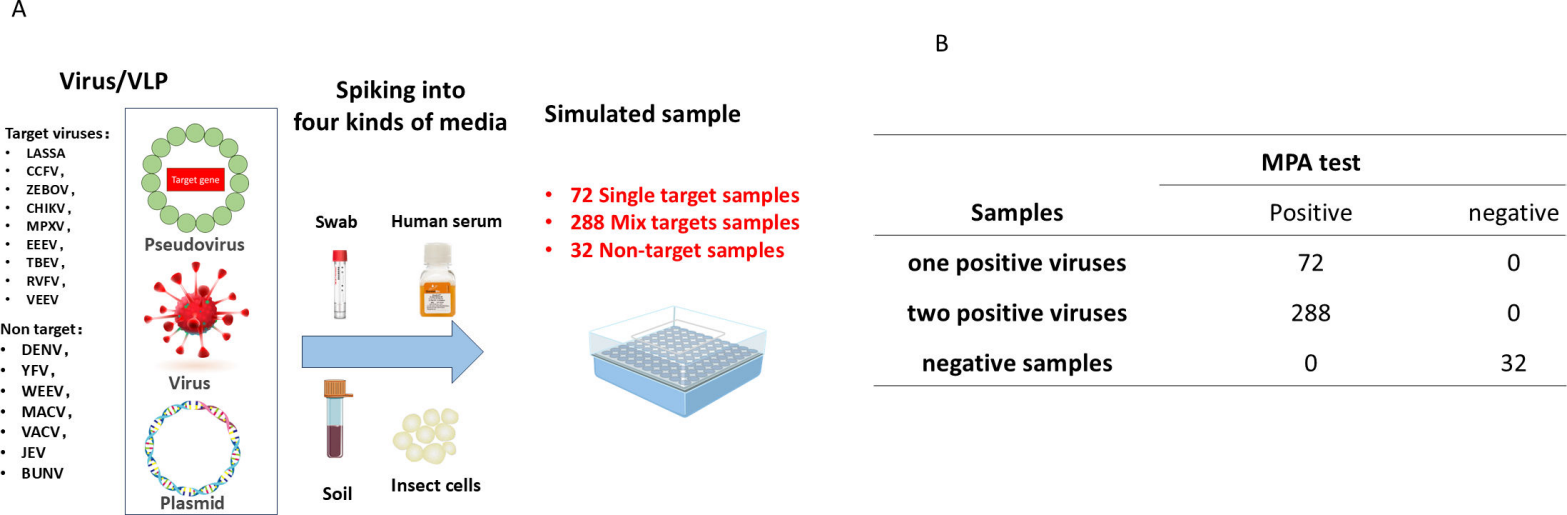
Validation of the MPA-nine-viruses by simulated samples. (A) Workflow diagram illustration of the simulated samples preparation and MPA test. (B) MPA test results. CCHFV, Crimean-Congo hemorrhagic fever virus; CHIKV, Chikungunya virus; EBOV, Ebola virus; EEEV, Eastern equine encephalitis virus; LASV, Lassa virus; MPXV, Monkeypox virus; RVFV, Rift Valley fever virus; TBEV, Tick-borne encephalitis virus; VEEV, Venezuelan equine encephalitis virus.

A total of 392 simulated samples of four types—serum, swabs, soil, and mosquito vectors—were prepared using medium and low concentrations of virus cultures or VLPs. The results, as shown in Fig. 5, revealed that among the 392 simulated samples, 72 were positive for a single pathogen, 288 were positive for two viruses, and 32 were negative. These results were entirely consistent with the actual viral genes contained in the samples.

### MPA-nine-viruses accurately detects clinical samples

**Fig 6:**
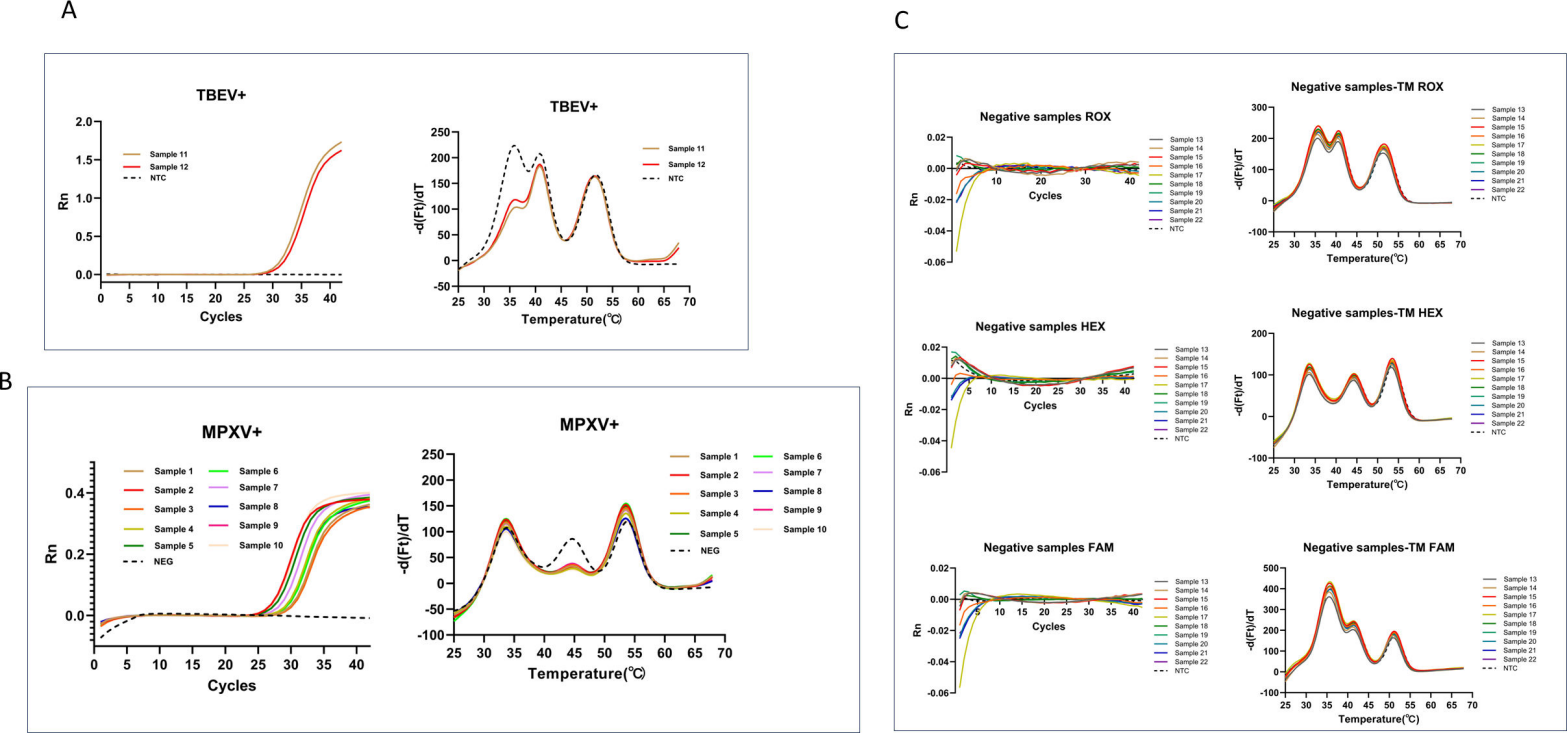
Validation of MPA-nine viruses for clinical samples. (A): MPA results of two TBE serum samples. (B) MPA results of ten TBE MPXV samples. (C) MPA results of ten negative samples. The negative control was in dotted line, and the samples were in solid.

The nine target viruses in this study are all highly infectious and lethal pathogens, most of the infections have not been reported in China. Currently, only a limited number of clinical samples are available for tick-borne encephalitis virus and MPXV. We utilized 10 MPXV-positive swabs, 2 Tick-borne encephalitis-positive serum samples, and 10 non-target samples (including 2 normal throat swabs, 2 normal serum samples, 4 influenza throat swab samples, and 2 COVID-19 patient samples) to validate the MPA-nine-viruses for clinical sample detection (Fig. 6).

The results demonstrated that the MPA accurately identified the viral nucleic acids in all 10 mpox patients and TBE patients. For the 10 mpox swabs, positive amplification curves appeared at the Hex channel, and the significant melting curve reduction appeared at *T*_m_ 45°C; for the 2 TBE serum samples, positive amplification curves appeared at the Rox channel, and the significant melting curve reduction appeared at *T*_m_ 36°C. These results were consistent with conventional qPCR results, and some CT values were even lower than those of traditional qPCR.

No positive signals were observed for all 10 negative samples at the FAM, HEX, or ROX channels indicate excellent specificity and sensitivity of the MPA-nine-viruses for clinical samples (Table 3). The positive signal of the internal control present in every sample at the Cy5 channel confirmed assay functionality and ruled out the possibility of false-negative results.

**TABLE 3:** Validation of MPA by 22 clinical samples^*a*^

Sample (No.)	MPA results							Traditional qPCR results	
	FAM		HEX		ROX		CY5 (Reference)	Virus	Virus (CT value)
	Ct	Tm	Ct	*T*_m_	Ct	*T*_m_	Ct		
1	NoCt	/	29.49	44.78	NoCt	/	33.09	MPXV	MPXV (30)
2	NoCt	/	29.68	44.78	NoCt	/	24.04	MPXV	MPXV (30)
3	NoCt	/	27.25	44.78	NoCt	/	24.64	MPXV	MPXV (30)
4	NoCt	/	28.53	44.78	NoCt	/	29.43	MPXV	MPXV (28)
5	NoCt	/	29.59	44.78	NoCt	/	34.06	MPXV	MPXV (29)
6	NoCt	/	27.1	44.78	NoCt	/	29.61	MPXV	MPXV (28)
7	NoCt	/	30.5	44.78	NoCt	/	35.65	MPXV	MPXV (32)
8	NoCt	/	30.27	44.78	NoCt	/	35.05	MPXV	MPXV (32)
9	NoCt	/	27.71	44.78	NoCt	/	30.72	MPXV	MPXV (29)
10	NoCt	/	29.74	44.78	NoCt	/	25.44	MPXV	MPXV (29)
11	NoCt	/	NoCt	/	30.65	35.55	30.05	TBEV	TBEV (31)
12	NoCt	/	NoCt	/	31.33	35.45	26.16	TBEV	TBEV (32)
13	NoCt	/	NoCt	/	NoCt	/	32	/	/
14	NoCt	/	NoCt	/	NoCt	/	33	/	/
15	NoCt	/	NoCt	/	NoCt	/	25	/	/
16	NoCt	/	NoCt	/	NoCt	/	27	/	/
17	NoCt	/	NoCt	/	NoCt	/	21	/	SARS-CoV-2 (29)
18	NoCt	/	NoCt	/	NoCt	/	21	/	SARS-CoV-2 (29)
19	NoCt	/	NoCt	/	NoCt	/	25	/	H1N1 (29)
20	NoCt	/	NoCt	/	NoCt	/	26	/	H1N1 (30)
21	NoCt	/	NoCt	/	NoCt	/	27	/	H1N1 (31)
22	NoCt	/	NoCt	/	NoCt	/	28	/	H1N1 (28)

^*a*^ "/" indicates that no *T*_m_ with significantly reduced melting peak.

## DISCUSSION

In this study, MPA-nine-viruses assay was established, which used three fluorescence channels (FAM, HEX, and ROX) and different melting curve temperatures to achieve simultaneous screening of nine viral targets in a single reaction tube. To ensure assay functionality and avoid false-negative results, a unique internal control sequence from the human genome was designed, labeled with Cy5, and incorporated into this MPA-nine-viruses assay. Thus, monitoring the presence of the internal control sequence was independent at the Cy5 channel of the presence or absence of target sequences in the assay. Therefore, a total of 10 gene targets were included in this assay: 9 target viruses and 1 internal reference gene.

In this MPA-nine-viruses assay, primers, THO, and PCO probes for 10 target genes are mixed together in the same tube, with a total of 39 primers or probes (no PCO probe for the internal reference gene). Dozens of primers and probes often interfere with each other and greatly affect the amplification efficiency. Therefore, it is crucial to design and select proper primer and probe sequences without interference; however, it is challenging (27). In this study, strict rules for primer-probe design were adopted, and the hairpin structure, dimer structure, GC%, and theoretical *T*_m_ value of PCO-THO were analyzed to theoretically ensure the candidate primers and probes met the requirements. After several rounds of design and experimental screening, the primers, THO, and PCO probes without interference were finally determined, which showed high sensitivity to all nine viral targets. The LODs were less than 15 copies/µL, and no cross-reaction was observed among the multiple targets. High specificity was also verified by using genes from other viruses, such as Yellow fever virus and Dengue virus. In addition, when multiple target genes exist simultaneously, even as many as nine targets, every target gene can be tested out, showing independent amplification efficiency without interfering with each other. The MPA-nine viruses would be reliable for the accurate detection of infected samples in future applications.

For validation of this MPA-nine-viruses in clinical use, since most of the target viruses were high pathogenic, both of nature virus strains and clinical samples were rare and very difficult to obtain, we used VLPs which contained the target RNA gene sequence and prepared multiple simulated samples: types of swabs, serum, soil, and mosquito cells; single target and mixed dual targets; medium (5 × 10^4^ gene copies/mL) and low concentration (5 × 10^3^ gene copies/mL); positive and negative samples. As many as 392 simulated samples were used for evaluation, all of which were accurately identified, indicating that the MPA developed in this study would potentially be applicable for clinical infection case testing and environmental monitoring. Furthermore, we collected 10 clinical swab samples from mpox patients, 2 serum samples from TBE patients, and 10 negative samples, totaling 22 clinical samples for testing. All target viral nucleic acids were accurately detected, and the CT values of the MPA were comparable to or even lower than those of conventional qPCR, which further verified the high sensitivity and accuracy of MPA for real clinical sample detection.

This MPA-nine-viruses utilizes the same instrument and 96-well plate as traditional qPCR, so this MPA-nine-viruses is low-cost and applicable for large-scale sample screening. With the advantages of high sensitivity, low cost, multiplex targets, and high-throughput screening, this MPA-nine-viruses would be potentially useful for the spread control and biothreat defense of related pathogens in the future.

However, as the detecting targets of this study are mainly biothreat viruses, most of these highly pathogenic viruses have not spread in China. Both the virus isolates and the clinical positive samples are rare and difficult to obtain. We only collected 12 positive clinical samples in this study, which were not sufficient for a thorough evaluation of this MPA-nine-viruses. This is a limitation of this study. Further evaluation by clinical samples is still needed, especially in the case of application approval by the FDA.

### Conclusion

In this study, we developed an MPA-nine-viruses method for simultaneously and accurately identifying nine highly pathogenic viruses in a single reaction, including Lassa fever virus, Ebola virus, Crimean-Congo hemorrhagic fever virus, Chikungunya virus, MPXV, Eastern equine encephalitis virus, Tick-borne encephalitis virus, Venezuelan equine encephalitis virus, Rift Valley fever revirus, and an internal reference gene, with up to 10 gene targets in a single reaction. The MPA-nine-viruses was highly sensitive and specific, with an LOD reaching 1.5–15 copies/µL for the nine viral target genes, and no cross-reaction among the nine viral targets. This MPA was validated by accurately testing 392 simulated samples and 22 clinical samples, including serum, swabs, mosquito cells, and soil, demonstrating the availability of MPA for multiple sample types and multiple viruses. With the advantages of multiple targets, high sensitivity, low cost, and high throughput, the MPA is expected to play an important role in the prevention and control of epidemics and terrorist attacks caused by related viruses in the future.

## Data Availability

All the data from the current study are shown in the manuscript and supplemental materials and are available online.

## SUPPLEMENTAL MATERIAL

Supplemental material spectrum.01078-25-s0001.docx Tables S1 and S2; Fig. S1.

ASM does not own the copyrights to Supplemental Material that may be linked to, or accessed through, an article. The authors have granted ASM a non-exclusive, world-wide license to publish the Supplemental Material files. Please contact the corresponding author directly for reuse.
