# Supplementary material for: Highly sensitive and multiplex detection of nine potential bioterrorism viral agents in a single reaction by multiplex probe amplification (MPA) with melting curve analysis
Source: Microbiol Spectr. 2025 Jul 31;13(9):e01078-25. doi: 10.1128/spectrum.01078-25 (PMC12403643; doi:10.1128/spectrum.01078-25)
Supplement: Supplemental material — Tables S1 and S2; Fig. S1. [file spectrum.01078-25-s0001.docx]

**Supplemental Table I.The selected target sequence、primers、THO and PCO probe for each virus.**

| Virus | Target | |
| --- | --- | --- |
|  | Sequence(5’-3’） | Gene |
| Lassa virus | GCGCACCGGGGATCCTAGGCATTTTTGGTTGCGCAATTCAAGTGTCCTATTTAAAATGGGACAAATAGTGACATTCTTCCAGGAAGTGCCTCATGTAATAGAAGAGGTGATGAACATTGTTCTCATTGCACTGTCTGTACTAGCAGTGCTGAAAGGTCTGTACAATTTTGCAACGTGTGG | E |
|  |  |  |
|  |  |  |
|  |  |  |
| Crimean-Congo hemorrhagic fever virus | CCACAGTGTTCTCTTGAGTGTTAGCAAAATGGAAAACAAAATCGAGGTGAATAACAAAGATGAGATGAACAAGTGGTTTGAAGAGTTCAAAAAGGGAAATGGACTTGTGGACACCTTCACAAACTCCTATTCCTTTTGTGAGAGTGTTCCAAATTTGGACAAGTTTGTATTCCAAATGGCCAGTGCCACTGATGATGCACAAAAGGATTCTATCTACGCGTCTGCTCTGGTGGAGGCAACAAAATTTTGTGCACCTATATATGAGTGTGCGTGGGTTAGCTCCACTGGCATTGTGAAGAGG | 3’NCR |
|  |  |  |
|  |  |  |
|  |  |  |
| Zaire Ebola virus | CCGAGTATGGATTCTCGTCCTCAGAAAATCTGGATGGCGCCGAGTCTCACTGAATCTGACATGGATTACCACAAGATCTTGACAGCAGGTCTGTCCGTTCAACAGGGGATTGTTCGGCAAAGAGTCATCCCAGTGTATCAAGTAAACAATCTTGAAGAAATTTGCCAACTTATCATACAGGCCTTTGAAGCAGGTGTTGATTTTCAAGAGAGTGCGGACAGTTTCCTT | NP |
|  |  |  |
|  |  |  |
|  |  |  |
| Chikungunya virus | CTGAGACTCCTACCATGCTGTTGTAAAATGTTGACTTTTTTAGCCGTGCTGAGCGTCGGTGCCCACACTGTGAGCGCGTACGAACACGTAACAGTGATCCCGAACACGGTGGGAGTACCGTATAAGACTCTAGTCAACAGACCGGGCTACAGCCCCATGGTACTGGAGATGGAG | 6k |
|  |  |  |
|  |  |  |
|  |  |  |
| Monkeypox virus | TAGGAGAGTTACTAGGCCCCACTGATTCAATACGAAAAGACCAATCTCTCCTAGTTATTTGGCAGTACTCATTAATAACGGTGACAGGGTTAACACCTTTCCAATAAATAATTTTTTTAACCGGAATAACATCATCAAAAGACTTATTATCCTCTCTCATTGATTTTTCGCGGGATACATCATCTATTATAGCATCAGCATCAGAATCTGTAGGCCGTGTATCAGCATCCATTGTCGTAGACCAACGAGGAGGAGTATCGTCGGAACTGTACACCATAGTACTACGTTGAAGATCATACAGAGCTTTATTAACTTCTCGCTTCTCCATATTAAGTTGTTTAGTTAGTTGTGCAGTAGCTCCTTAGTCCAATGTTTTTAATAACCGCACACGATTACTACATTATTAAATCATGAGGTCCGTATTATACTCGTATATATTGTTTCTCTCATGTATAATAATAAACGGAAGAGATATAGCACCACATGCACCATCCAATGGAAAATGTAAAGACAACGAATACAGAAGCCGTAATCTATGTTGTCTATCGTGTCCTCCGGGAACTTACGCTTCCAGATTATGTGATAGCAAGACTAATACACAATGTACGCCGTGTGGTTCGGATACCTTTACATCTCACAATAATCATTTACAGGCTTGTCTAAGTTGTAACGGAAGATGTGATAGTAATCAGGTAGAGACGCGATCGTGTAACA | Double-stranded RNA binding protein |
|  |  |  |
|  |  |  |
|  |  |  |
| Eastern equine encephalitis virus | CCGATGGTCCAACCTGAAAGGTTGGTGCCTAGGCGACCTGCACCGCCTGTGCCCGTACCTGCAAGAATCCCCAGCCCTCCATGTACATCGACCAATGGATCGACGACCAGTATACAATCACTGGGGGAGGATCAAAGCGCATCTGCTTCTAGCGGAGCTGAAATCTCTGTAGACCAGGTTTCGCTATGGAGCATACCCAGCGCTACCGGGTTCGATGTGCGTACCTCCTCATCGTTGAGCCTAGAGCAGCCTACCTTTCCGACAATGGTTGTCGAAGCAGAGATTCACGCCAGTCAAGGA | NSP3 |
|  |  |  |
|  |  |  |
|  |  |  |
| Tick borne encephalitis virus | GAGAAATTGGCAGCTCCTTTCAGGATTTTTCCTCCTCCTATACTAAATTCCCCCTCAATAGAGGGGGGGCGGTTCTTGTTCTCCCTGAGCCACCATCACCCAGACACAGATAGTCTGACAAGGAGGTGACGTGTGACTCGGA | NS1 |
|  |  |  |
|  |  |  |
|  |  |  |
| Rift Valley fever virus | TTGTTCAATCGGTGGTGGAGACAATAGCCAGGTCCATAGGGAAGTTCTTTGCTTCTGATACCCTCTGTAACCCCCCCAATAAAGTGAAAATTCCTGAGACACATGGCATCAGGGCTCGGAAGCAATGTAAGGGGCCTGTGTGGACTTGTGCAACATCAGATGATGCAAGGAAGTGGAACCAAGGCCATTT | polymerase |
|  |  |  |
|  |  |  |
|  |  |  |
| Venezuelan equeine encephalitis virus | CATGGAGGTCAAGATCATAGACGCTGTGGTGGGCGAGAAAGCGCCCTATTTCTGTGGAGGGTTTATTTTGTGTGACACCGTGACCGGCACAGCGTGCCGTGTGGCAGACCCCCTAAAAAGGCTATTTAAGCTTGGCAAACCTCTGGCGGCAGATGATGAACATGACGATGACAGGAGAAGGGCATTACACGAAGAGTCAACACGCTGGAATCGAGTGGGAATTCTTCCAGAGCTGTGTAAGGCAGTAGAATCAAGGTATGAAACCGTAGGAACTTCCATCATAGTTATGGCCATGACTACTCTAGCTAGCAGTGTTAAATCATTCAGCTACCTGAGAGGGGCCCCTATAACTCTCTACGGCTAACCTGAA | NP |
|  |  |  |
|  |  |  |
|  |  |  |

**Supplemental Table2.The selected primers、THO and PCO probe for each virus.**

| Virus | Primer/Probe | Sequence（5’-3’） |
| --- | --- | --- |
| Lassa virus | LASV-F | GCGCAATTCAAGTGTCCT |
|  | LASV-R | AGTAYRGACAGTGCAATGAGA |
|  | LASV-THO | FAM-TCATCACCTCTTCTATYACATGAGGYACTTCCT-BHQ1 |
|  | LASV-PCO | AGGAGGTTCCTAATGTCATATAAGATGTGGTGA-Phosphate |
| Crimean-Congo hemorrhagic fever virus | XHFV-F | GCARAATGGAAAACAARATCGAGGT |
|  | XHFV-R | CCAYAAGTCCATTTYCCTTTTTRAA |
|  | XHFV-THO | FAM-TAACAARGATGAGATGAACARGTGGTTTGAAGAG-BHQ1 |
|  | XHFV-PCO | CTCTTCAAACCACATATTTATATCATCGTTGTTA-Phosphate |
| Ebola virus | EBO- F | AGCAGGTCTGTCTGTTCAACA |
|  | EBO- R | GGCCTGTATGATAAGTTGGCAAA |
|  | EBO- THO | FAM-TCGGCAAAGAGTCATCCCAGTGTATCAA-BHQ1 |
|  | EBO-PCO | TTGATACAATGAGATAACTATTTGTCGA-Phosphate |
| Chikungunya virus | CHV-F | TCCTACCATGCTGTTGTAAAATGTTG |
|  | CHV-R | AGAGTCTTATACGGTACTCCCAC |
|  | CHV-THO | VIC-TGAGCGTCGGTGCCCACACTGTGA-BHQ1 |
|  | CHV-PCO | TCGCAGAGTTGACACAGATGCTCA-Phosphate |
| Monkeypox virus | mpox-F | CTCTCATTGATTTTTCGCGGGATA |
|  | mpox-R | CGATACTCCTCCTCGTTGGTC |
|  | mpox-THO | VIC-CATCAGCATCAGAATCTGTAGGCCGTGT-BHQ1 |
|  | mpox-PCO | ACACGTCCTACATACTCTAATGCTGATG-Phosphate |
| Eastern equine encephalitis virus | EEEV-F | CCAAYGGATCGACGACCAGTAT |
|  | EEEV-R | AGAGAKTTCAGCTCCGCTAGAAG |
|  | EEEV-THO | VIC-CAGATGCGCTTTGATCCTCCCCCAGTG-BHQ1 |
|  | EEEV-PCO | CATTGGTGGAGGATCGAAGCTCATCTG-Phosphate |
| Tick borne encephalitis virus | TBEV-F | GGGCGGTTCTTGTTCTC |
|  | TBEV-R | CATCACCTCCTTGTCAGACT |
|  | TBEV-THO | ROX-TGAGCCACCATCACCCAGACACA-BHQ2 |
|  | TBEV-PCO | TGTGTCTGTGTTATGTTGTCTCA-Phosphate |
| Rift Valley fever virus | RVFV-F | AGTTCTTTGCKTCTGATACCCTCTG |
|  | RVFV-R | TCTGATGTWGCACAAGTCCAC |
|  | RVFV-THO | ROX-CCCCTTACATTGCTTCCGRGCCCTGATG-BHQ2 |
|  | RVFV-PCO | CATCAGGACACGTAAGTAATGTAAGGGG-Phosphate |
| Venezuelan equeine encephalitis virus | VEEV-F | ACAGGAGAAGGGCATTRCAYGA |
|  | VEEV-R | AAGTTCCTACGGTTTCATACCTTGAT |
|  | VEEV-THO | ROX-ACACAGCTCTGRAAGAATTCCCACTCGAT-BHQ2 |
|  | VEEV-PCO | ATCGAGTGAGACTTATTGCAGATCTGTGT-Phosphate |


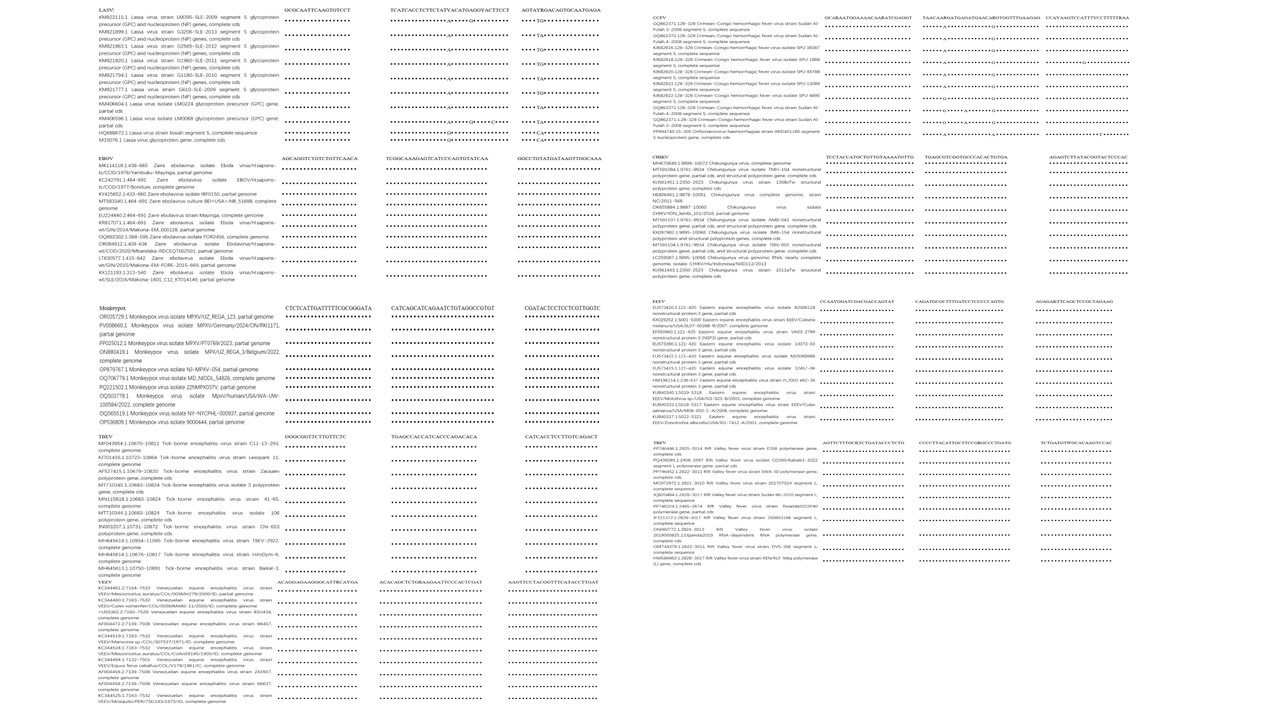


Sup. Fig. 1 Alignment of primer and THO with target genes from different stains.
